# Evaluation of TV commercials using neurophysiological responses

**DOI:** 10.1186/s40101-015-0056-4

**Published:** 2015-04-24

**Authors:** Taeyang Yang, Do-Young Lee, Youngshin Kwak, Jinsook Choi, Chajoong Kim, Sung-Phil Kim

**Affiliations:** Department of Human and Systems Engineering, Ulsan National Institute of Science and Technology, 50 UNIST-gil, Eonyang-eup, Ulju-gun, Ulsan, 689-798 Korea; Division of General Studies, Ulsan National Institute of Science and Technology, 50 UNIST-gil, Eonyang-eup, Ulju-gun, Ulsan, 689-798 Korea; Department of Industrial Design, Ulsan National Institute of Science and Technology, 50 UNIST-gil, Eonyang-eup, Ulju-gun, Ulsan, 689-798 South Korea

**Keywords:** Neurophysiological indices, Electroencephalography (EEG), TV commercials, Advertising

## Abstract

**Background:**

In recent years, neuroscientific knowledge has been applied to marketing as a novel and efficient means to comprehend the cognitive and behavioral aspects of consumers. A number of studies have attempted to evaluate media contents, especially TV commercials using various neuroimaging techniques such as electroencephalography (EEG). Yet neurophysiological examination of detailed cognitive and affective responses in viewers is still required to provide practical information to marketers. Here, this study develops a method to analyze temporal patterns of EEG data and extract affective and cognitive indices such as happiness, surprise, and attention for TV commercial evaluation.

**Methods:**

Twenty participants participated in the study. We developed the neurophysiological indices for TV commercial evaluation using classification model. Specifically, these model-based indices were customized using individual EEG features. We used a video game for developing the index of attention and four video clips for developing indices of happiness and surprise. Statistical processes including one-way analyses of variance (ANOVA) and the cross validation scheme were used to select EEG features for each index. The EEG features were composed of the combinations of spectral power at selected channels from the cross validation for each individual. The Fisher’s linear discriminant classifier (FLDA) was used to estimate each neurophysiological index during viewing four different TV commercials. *Post hoc* behavioral responses of preference, short-term memory, and recall were measured.

**Results:**

Behavioral results showed significant differences for all preference, short-term memory rates, and recall rates between commercials, leading to a ‘high-ranked’ commercial group and a ‘low-ranked’ group (*P* < 0.05). Neural estimation of happiness results revealed a significant difference between the high-ranked and the low-ranked commercials in happiness index (*P* < 0.01). The order of rankings based on happiness and attention matched well with the order of behavioral response rankings. In the elapsed-time analysis of the highest-ranked commercial, we could point to visual and auditory semantic structures of the commercial that induced increases in the happiness index.

**Conclusions:**

Our results demonstrated that the neurophysiological indices developed in this study may provide a useful tool for evaluating TV commercials.

## Background

Neuroscientific findings have helped us to understand how the brain encodes and represents the environment and how it controls our body [1]. Several non-invasive tools to measure neurophysiological responses have been well developed and widely used such as electroencephalography (EEG), magnetoencephalography (MEG), and functional magnetic resonance imaging (fMRI) [2,3]. Advances in neuroscience have settled into our daily lives, social practices, and even academic discourses. Frazzetto and Anker [4] called this trend ‘Neuroculture.’ Various research fields have adopted neurotechnologies and acquired a ‘neuro’ dimension [4], such as neuroeconomics [5], neurotheology [6], and neuroeducation [7,8].

Marketers have also sought to understand the complex process of evaluation and buying in the human brain [9]. Conventional assessment tools to measure the effects of marketing depend largely on personal evaluation and market analysis. These methods scarcely recognize causal interactions between marketing strategies and their impacts on consumers’ cognitive and affective responses. In addition, these *post hoc* analysis methods are often influenced by the participants’ mental state or environment at the time of the survey. Marketers, therefore, attempt to supplement these drawbacks and explore tactics and technologies that affect decision makers unconsciously [9].

According to Ariely and Berns [10], neuroscientific tools could be attractive for a marketer in two ways: first, they are cheaper and faster than current marketing methods, and second, they offer detailed information about product evaluation that conventional marketing methods cannot disclose. As a result, neuromarketing, where neuroscience and marketing meet, has emerged [11] as the application of neuroscientific methods to analyze and understand human behavior in relation to markets and marketing exchanges [12]. Although this field has been controversial since its birth in 2002, it has received credibility and has been adopted by many advertising and marketing professionals, thereby growing rapidly [2]. Neurophysiological methods, unlike conventional methods, can offer the perspectives of a diverse range of methods to evaluate the effectiveness of products, brand logos, and commercial videos before starting the actual advertising campaign [11]. A number of neurophysiological studies have evaluated preferences for products in terms of brand familiarity [3,13-16]. The current research consists of ‘user design,’ a new trend that lets consumers participate in the design of new products and involves not only what consumers express but also what they think [10].

In neuromarketing and other neuroculture fields, recognition of the emotional states of users (or customers) has become one of the most important themes. Affect has played an important role in rational and intelligent behavior as well as in communication between humans [17]. Accordingly, studies seeking to identify brain regions and frequency bands most closely related to various emotional states are needed.

There have been several studies, especially within the last decade, that have investigated emotion recognition and media evaluation. For example, Lin and his colleagues classified emotional states (joy, anger, sadness, and pleasure) from EEG dynamics together with self-reported emotional states while listening to music [18,19]. Wang *et al*. [20] designed a classification system for four emotional states (joy, relax, sadness, and fear) with EEG using movie stimuli. Kwon and his colleagues also proposed an emotion-recognition system from movie clips using EEG. Emotions were classified into positive or negative affections with alpha and gamma power-band EEG features [21,22].

A number of neurophysiological studies have also attempted to identify cognitive and affective indices of emotion by evaluating TV contents using EEG. Reeves *et al*. [23] found that the alpha band activity (interval = 0.5 s) for commercial videos had significant negative correlations with recall and recognition of their contents. Reeves *et al*. [24] reported a significant interaction between hemisphere and emotional content of TV commercials for the frontal alpha but no interaction for the occipital alpha. Another study on the relationship between viewers’ attention and semantically related TV sequences showed that related and unrelated sequences yielded significant differences in reaction time [25]. Simons *et al*. [26] studied a correlation between the emotional content of TV commercials and viewers’ attention and reported that the emotional content affected the alpha power wave in the parietal region. Smith and Gevins [27] investigated the changes in alpha waves in relation to changes in video structure (for example, scene change rate) and semantic structure (for example, emotional impact of scene) and found that recall rate improved as the occipital alpha waves decreased when the commercial was presented at a higher scene change rate. Bertin *et al*. [28] evaluated TV commercials for the *Asahi Shimbun* newspaper with EEG and showed correlations between prefrontal cortical activity and survey-based evaluations.

Most previous neurophysiological studies on TV commercials have sought for neural correlates of various contents and evaluations of TV commercials. However, marketers may need much diverse information from neurophysiological research than just finding neural correlates for practical applications. For instance, it would be useful if one can assess which parts of a TV commercial elicit positive emotional responses or attract more attention in viewers. Therefore, the present study aims to develop a neurophysiological method to predict the temporal patterns of cognitive and affective states of viewers during watching TV commercials from EEG. Two hypotheses of the present study are formed as follows: (1) the model-based neurophysiological indices developed using non-commercial stimuli can be applied to evaluate TV commercials and consistent with subjective behavioral responses. (2) The temporal patterns of the neurophysiological indices may be correlated with the important scenes of the commercials.

Assessment of cognitive and affective states is designed based on three indices - happiness, surprise, and attention. In addition, to address a problem of inter-individual variations in EEG in response to the same video stimulus, we build a model-based assessment scheme where the classification model translates individual EEG patterns into normative scores for each index. To validate the neurophysiological analysis results, we also evaluate participants’ behavioral responses in terms of preference, short-term memory, and recall and compare them with neurophysiological indices. Finally, we investigate a feasibility of extracting parts of TV commercials that induce positive emotional responses in viewers using our model.

## Methods

### Ethics statement

This investigation was conducted according to the principles expressed in the Declaration of Helsinki. All participants provided informed written consent prior to participation with pre-approval obtained from the Institutional Review board of the Ulsan National Institute of Science and Technology (UNISTIRB-14-01-C).

### Experimental stimuli

Two sets of video stimuli and one game stimulus were used in this experiment. The first set of video stimuli were used to measure basic emotional states for the development of a basic neurophysiological index assessment model, while the second set consisting of TV commercials was used to evaluate our model. The game stimulus was used to focus participants’ attention.

**Figure 1:**
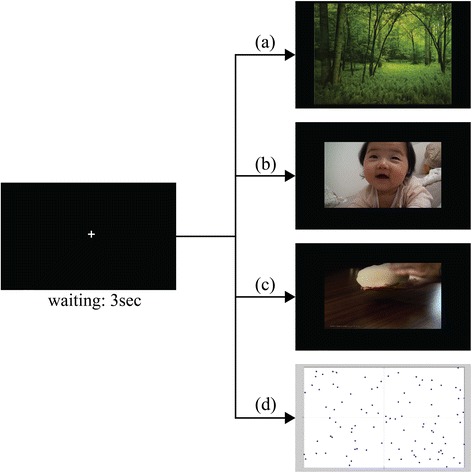
Method to present each stimuli. Process to present visual stimuli. For fixation, a white cross on black background appear on the screen for 3 s. Top three pictures on right side are samples of video used to induce responses of **(a)** neutral, **(b)** happiness, and **(c)** surprise. **(d)** Last image shows a game to maintain the attention of participants.

For the TV commercial evaluation index development, there were four types of video stimuli. First, a neutral video containing green-colored scenery and nature sounds was used as a baseline neutral stimulus. Second, two videos were presented to induce emotional responses and to develop a happiness index in each participant; they presented the 2002 World Cup Korean soccer game (H1) or a dozing and smiling baby (H2). For the analysis, we selected the more appropriate video among these two videos depending on participants’ verbal responses after viewing. Third, another pair of video clips was presented to induce emotional responses of surprise - these showed a new way to pour beer (S1) or magnetic sand (S2). Again, one was selected based on the participants’ responses. Fourth, a moving dot game was used to focus participants’ attention [29]. In this game, participants had to identify a dot moving at a faster speed than the other dots, which moved at a constant speed and direction. During the game, participants were asked to indicate the dot’s location by pressing one of four keyboard buttons (Q, W, A, or S) mapped onto the quadrants of the screen (Figure 1). To fully engage their attention in the game, participants were informed that they would receive an extra reward if they achieved the highest score.

The second set of video stimuli, used for the TV commercial evaluation, was selected from smartphone commercials released after 2013. Six commercials from different brands were selected; they all had similar running times (approximately 30 s). We excluded two commercials from the most widely used brands (in Korea) to reduce the influence of attachment to a particular brand. Accordingly, four commercials were used in this experiment (C1, C2, C3, and C4). Each commercial has different contents and delivers the messages in different ways. For instance, C1 emphasized the function of the rear button using the metaphor by showing many couples with the concealed present box behind men. Then, a message follows as, ‘We always hide what is precious behind us.’ C2 stressed the function for taking a picture and color variation with animated atmosphere in the kitchen in the morning. C3 was a typical informative commercial, delivering their design and functional aspect only without any affective scenes. C4 demonstrated their characteristics such as ‘oscillatory sound’ with the scene in the club. The volume of the music was frequently changed according to its feature. We collected each commercial’s data from a publically available database (http://www.tvcf.co.kr/).

### Participants and experimental tools

Ten men and ten women (mean age 22.9 ± 1.41 years) with normal or corrected-to-normal vision and who did not report any neurological disorders participated in this study. During the experiment, scalp EEG signals were recorded using a wireless EEG headset (EPOC, Emotiv Inc., San Francisco, USA) from 14 electrodes located at AF3, F7, F3, FC5, T7, P7, O1, O2, P8, T8, FC6, F4, F8, and AF4 (in accordance with the International 10/20 system). EEG signals were sampled at 128 Hz and referenced to the average of the common mode sense (CMS, active electrode) and driven right leg (DRL, passive electrode) electrodes. The voltage between electrodes and the reference was amplified by 60 to 100 dB of voltage gain. The impedance levels of all of the electrodes were kept below 5 kΩ. All visual stimuli in this experiment were presented on a 27-in. monitor (QH270-IPSM, Achieva Korea, Incheon, Korea) positioned at about 60 cm distance from the participants’ eyes. Auditory stimuli were presented via a speaker positioned in the center. During the moving dot game, each participant used the keyboard to perform the task (that is, button press). We also used a questionnaire for the cognitive assessment and to collect behavioral responses to the presented commercials. This questionnaire originated from previous research [30] but was modified for the purpose of this study. We examined each participant’s preference for the commercials, brand name, and their degree of purchase intention. The preference for commercials was assessed by ranking each commercial. The preference for brand name and the degree of purchase intention were assessed using the Likert 7-point scale (1 ~ 7). In addition, participants were asked to answer whether they had seen a particular commercial or not, if they had known the brand name or not, and if they had previously purchased products of a particular brand or not. Finally, they were asked to determine the ranking of commercials based on how well they remember. In the next day, participants determined the ranking of commercials again based on how well they recall them after 1 day. In this study, we selected the preference for commercials, the degree of short-term memory, recall rate, and the degree of purchase intention for further analysis. All data were analyzed using the MATLAB program (Mathworks, Natick, MA, USA).

### Experimental procedure

**Figure 2:**
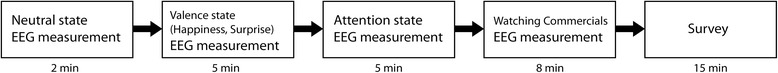
The experimental process. The experiment was conducted with five sessions. In first four sessions, EEG was recorded for neural response. In the final session, questionnaire was used to measure cognitive and affective response and develop preference, short-term memory and recall rate indices.

Before the experiment, each participant was situated in a comfortable chair for a few minutes. The EEG apparatus was installed during this time, and a brief explanation about the experiment was given. The experimental procedure consisted of five sessions in total, with four sessions for the EEG recording and one session for the questionnaire (Figure 2). In the first session, the green scenery picture with the nature sounds was presented to the participants for the neutral state condition. In the second session, we used four emotion-inducing video stimuli (two for happiness and two for surprise) to elicit the corresponding emotions from the participants. They were asked to indicate their emotion immediately after viewing each video clip to confirm that the evoked emotion matched our expectation. Participants selected their experienced emotion among seven emotional states (Ekman’s six basic emotions [31] plus not being classified). The rate of emotional feeling for each stimulus was 95% for H1, 50% for H2, 55% for S1, and 65% for S2 (H1: happiness stimulus 1, H2: happiness stimulus 2, S1: surprise stimulus 1, and S2: surprise stimulus 2). From these, we selected H1 and S2 as our basic stimuli.

In the third session, participants played the moving dot game, which sought to focus their attention. Participants were motivated by the promise of extra rewards for achieving the highest accuracy and completing the game in the shortest amount of time. In the fourth session, each of four commercials was presented randomly and participants simply viewed the commercials. In the final session, participants completed the questionnaire to evaluate each commercial. They ranked their preferences across all commercials and were asked to write down the specific products advertised in the commercials from memory to evaluate the short-term memory index of each commercial. Each ranking was converted to 4, 3, 2, and 1 points as the first, second, third, and fourth order, respectively. One day after the experiment, participants ranked how much they remembered from each commercial to build up a recall rate index.

### Feature extraction

EEG signals from 18 participants were further analyzed, as two participants’ data were contaminated by external noise. The EEG signals were band-pass filtered (0.5 to 100 Hz) and re-referenced to a common average reference to reduce potential shifts due to external artifacts. Each EEG data was split into quarter-second windows without overlap. EEG data in each window were analyzed at six frequency bands using short-time Fourier transform (STFT): delta (0.5 to 4 Hz), theta (4 to 8 Hz), alpha (8 to 12 Hz), low beta (12 to 20 Hz), high beta (20 to 30 Hz), and gamma (30 to 50 Hz). In each frequency band, the power spectral density from the spectrogram was averaged (frequency interval of 0.5 Hz). These steps extracted a total of 84 features from 14 channels and 6 frequency bands for each time window.

### Feature selection

One-way analyses of variance (ANOVA) and cross-validation were used to identify the optimal feature set for each participant’s cognitive/affective state classification models. Initially, 84 features were analyzed with separate one-way ANOVAs. Neural features collected for each of happiness, surprise, and attention were compared with those collected from the neutral session. From the results of ANOVAs, the first optimal feature with the highest *F-*value was selected. Along with this first feature, the next feature was selected with which classification accuracy through a tenfold cross-validation was maximized. This subsequent feature selection procedure based on tenfold cross-validation was repeated until its accuracy began to decrease. This process was performed for each participant and each neurophysiological index. Accordingly, the sizes of the optimal feature set differed between participants and neurophysiological indices. Finally, we obtained three EEG feature sets for each participant: neutral-happiness, neutral-surprise, and neutral-attention.

### Classification analysis

Upon finding the optimal feature set for each cognitive or emotional state, the state classifier model was trained to classify brain signals into one of two states: neutral *vs*. happiness, neutral *vs*. surprise, or neutral *vs*. attention. The Fisher’s linear discriminant classifier (FLDA) is a well-known classification method to determine an optimal hyper-plane to separate the data space according to the classes [32,33]. The FLDA aims to determine a projection vector, *w*, to maximize between-class scatter and to minimize within-class scatter. The Fisher criterion function, *J*(*w*),is then defined as follows:

1 $$ J(w)\kern0.5em =\kern0.5em \frac{w^T{S}_Bw}{w^T{S}_ww} $$

where:

2 $$ {S}_B\kern0.5em =\kern0.5em {\displaystyle \sum_c\left({\mu}_c\kern0.5em -\kern0.5em \overline{x}\right)}\kern0.5em {\left({\mu}_c\kern0.5em -\kern0.5em \overline{x}\right)}^T $$

is a between-class scatter matrix, and,

3 $$ {S}_w\kern0.5em =\kern0.5em {\displaystyle \sum_c{\displaystyle \sum_{i\in c}\left({x}_i\kern0.5em -\kern0.5em {\mu}_c\right)}}\kern0.5em {\left({x}_i\kern0.5em -\kern0.5em {\mu}_c\right)}^T $$

is a within-class scatter matrix. *c* denotes the class label and *μ*_*c*_ does the mean of data from the class *c*. $\overline{x}$ denotes the mean of the data from all the classes. The projection vector (*w*) is the vector that maximizes *J*(*w*).

The trained classifiers were used to estimate state variations during watching TV commercials. The EEG signals for each commercial were divided into a series of segments (window length: 0.25 s), and the features sets were selected for each segment. The classifiers were applied to the features, yielding a posterior probability of estimating a particular cognitive or emotional state. These posterior probabilities were used as neurophysiological indices for temporal patterns of happiness, surprise, and attention. The average of these indices were calculated and compared across the commercials. Finally, the commercials were subjected to an elapsed-time analysis in terms of the scene and auditory structures.

## Results

### Optimal feature set

**Figure 3:**
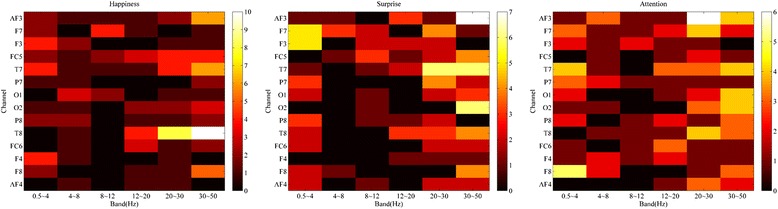
The counted number of each feature (frequency band - channel). The accumulated number of each feature by neurophysiological index (left: happiness, mid: surprise, right: attention). The brighter the cell is, the more frequently the feature is selected.

We examined how many times each feature (a combination of frequency and channel) was selected across all participants. Figure 3 shows the accumulated number of selecting each feature. It shows that EEG features with the high frequencies were more likely to be selected for the happiness and surprise indices.

**Figure 4:**
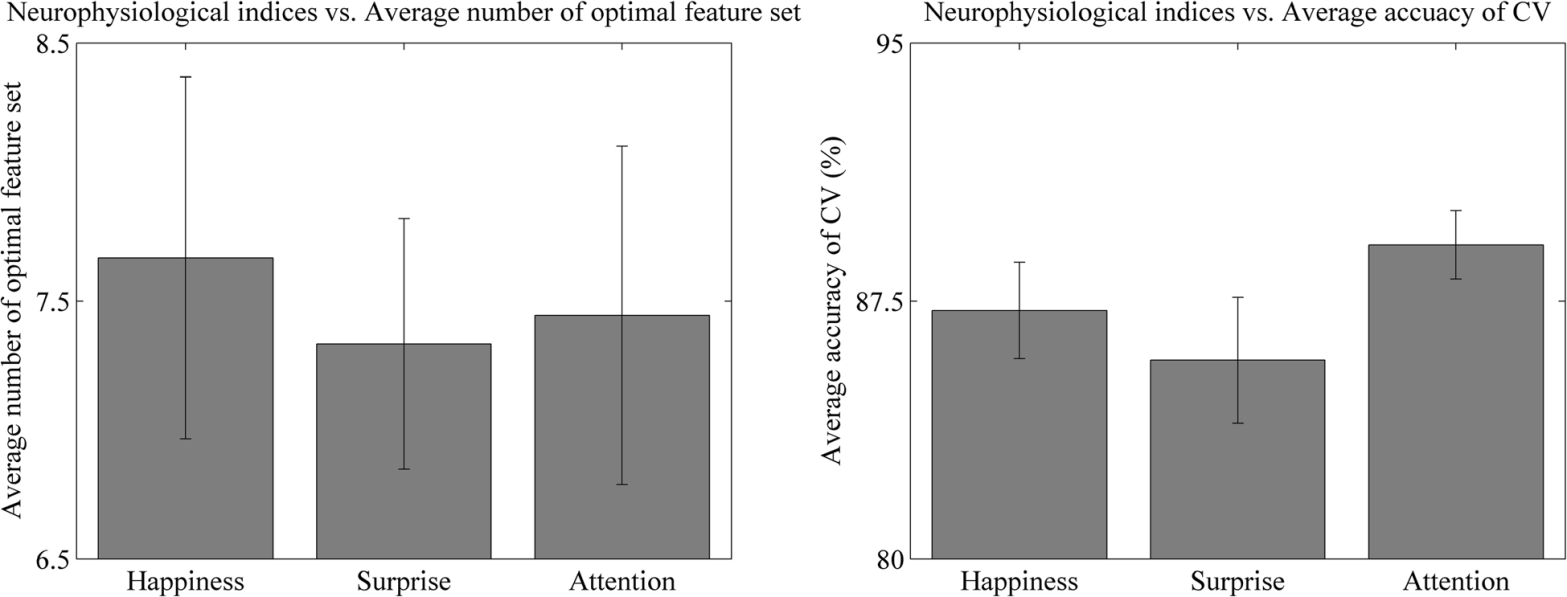
Average number of feature set and accuracy of cross validation. (Left) average number of optimal feature set (feature dimension) (right) average accuracy of cross validation. The number of features was 7.67 ± 0.70 (happiness), 7.33 ± 0.49 (surprise), and 7.44 ± 0.66 (attention). Average accuracy was 87.22% ± 1.40% (happiness), 85.77% ± 1.8% (surprise), and 89.13% ± 0.96% (attention). CV, cross validation.

We also compared the average of feature dimensions and cross-validation accuracy across neurophysiological indices. Figure 4 shows the average number of optimal feature sets and the accuracy of the cross-validation among the three neurophysiological indices. The number of features was 7.67 ± 0.70 for happiness, 7.33 ± 0.49 for surprise, and 7.44 ± 0.66 for attention. Average cross-validation accuracy was 87.22% ± 1.40% for happiness, 85.77% ± 1.8% for surprise, and 89.13% ± 0.96% for attention. There were no significant differences in the number of features or cross-validation accuracy between neurophysiological indices (*P* > 0.05).

### Behavioral responses

**Figure 5:**
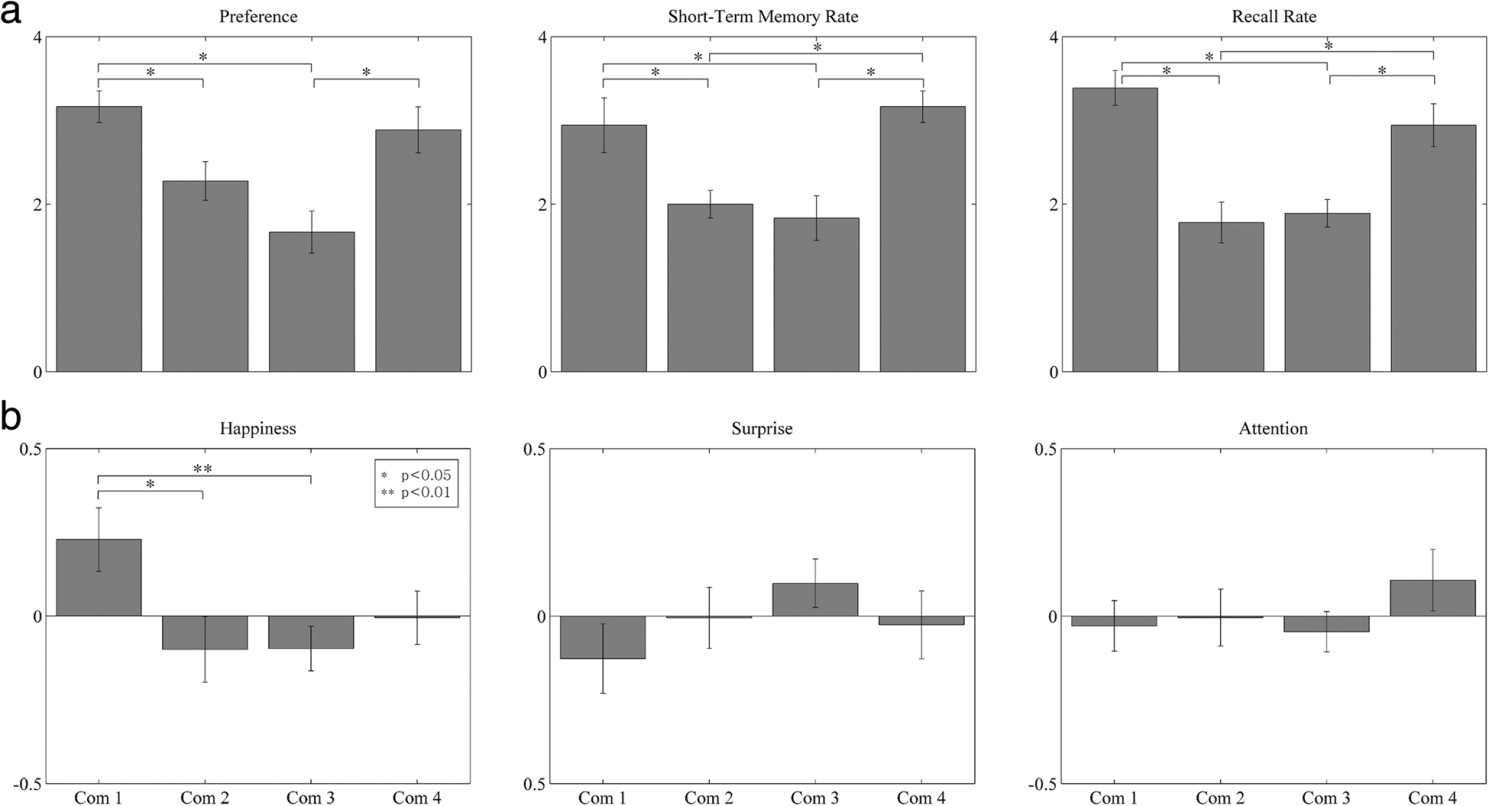
Statistical analysis of behavioral and neural data. Bar plot of analysis of each commercials in terms of **(a)** behavioral responses (preference rate, short-term memory rate, and recall rate) and **(b)** neurophysiological indices (happiness, surprise, and attention). *P < 0.05 and **P < 0.01. Com, commercial video.

The *post hoc* survey results showed that the preference rating was 3.17 ± 0.79 for C1, 2.28 ± 0.96 for C2, 1.67 ± 1.03 for C3, and 2.89 ± 1.13 for C4. Also, the short-term memory rate was 2.94 ± 1.35 for C1, 2.00 ± 0.69 for C2, 1.83 ± 1.10 for C3, and 3.17 ± 0.79 for C4; and the recall rate was 3.39 ± 0.85 for C1, 1.78 ± 1.00 for C2, 1.89 ± 0.68 for C3, and 2.94 ± 1.06 for C4. The statistical analysis results showed that the preference rating of C1 was higher than those of C2 and C3 (*P* < 0.05) and the preference rating of C4 was higher than that of C3 (*P* < 0.05). Also, the short-term memory rates of both C1 and C2 were higher than those of both C3 and C4 (*P* < 0.05). Similarly, the memory recall rates of both C1 and C2 were higher than those of both C3 and C4 (*P* < 0.05). No difference of the behavioral responses between C1 and C4 was found. Figure 5a illustrates a comparison of the behavioral responses (preference rating, short-term memory rate, and recall rate) for the four commercials (C1, C2, C3, and C4). The rankings of each index were determined as C1 > C4 > C2 > C3 for the preference rating, C4 > C1 > C2 > C3 for the short-term memory rate, and C1 > C4 > C3 > C2 for the recall rate. Participants were also asked to respond their purchase intention on each commercial. The scale was 1 (never purchase) to 7 (certainly purchase). The average rating was 3.72 ± 1.53 for C1, 2.17 ± 1.25 for C2, 1.83 ± 1.47 for C3, and 3.61 ± 1.69 for C4. The ranking of commercials in terms of the degree of purchase intention was similar to those of other behavioral responses and neurophysiological indices; C1 and C4 were significantly higher than both C2 and C3.

### Neurophysiological indices

Figure 5b shows a comparison of the neurophysiological indices (happiness, surprise, and attention) between the four commercials. The indices were normalized to values between −1 and 1, where larger positive numbers indicate that the corresponding emotion was induced more strongly. Happiness index was 0.23 ± 0.39 for C1, −0.1 ± 0.4 for C2, −0.09 ± 0.27 for C3, and −0.01 ± 0.33 for C4. Surprise index was −0.13 ± 0.43 for C1, −0.01 ± 0.38 for C2, 0.10 ± 0.30 for C3, and −0.03 ± 0.42 for C4; and attention index was −0.03 ± 0.31 for C1, 0.00 ± 0.35 for C2, −0.05 ± 0.25 for C3, and 0.11 ± 0.38 for C4. The happiness index of C1 was greater than those of C2 (*P* < 0.05) and C3 (*P* < 0.01). Besides this, there was no significant difference between any pair of indices. The ranking with the averages of each index was C1 > C4 > C3 > C2 for happiness, C3 > C2 > C4 > C1 for surprise, and C4 > C2 > C1 > C3 for attention, respectively (Table 1).

**Table 1:** **Means and standard deviations of each score and indices**

		**Com1**	**Com2**	**Com3**	**Com4**
Score in database (0 ~ 5)		3.77	3.12	2.91	3.57
Purchase intention (1 ~ 7)		3.72 ± 1.53	2.17 ± 1.25	1.83 ± 1.47	3.61 ± 1.69
Behavioral responses (0 ~ 4)	Preference	3.17 ± 0.79	2.28 ± 0.96	1.67 ± 1.03	2.89 ± 1.13
	Short-term memory	2.94 ± 1.35	2.00 ± 0.69	1.83 ± 1.10	3.17 ± 0.79
	Recall rate	3.39 ± 0.85	1.78 ± 1.00	1.89 ± 0.68	2.94 ± 1.06
Neurophysiological indices (−1 ~ 1)	Happiness	0.23 ± 0.39	−0.1 ± 0.4	−0.09 ± 0.27	−0.01 ± 0.33
	Surprise	−0.13 ± 0.43	−0.01 ± 0.38	0.10 ± 0.30	−0.03 ± 0.42
	Attention	−0.03 ± 0.31	0.00 ± 0.35	−0.05 ± 0.25	0.11 ± 0.38

Com, commercial video.

### Elapsed-time analysis

**Figure 6:**
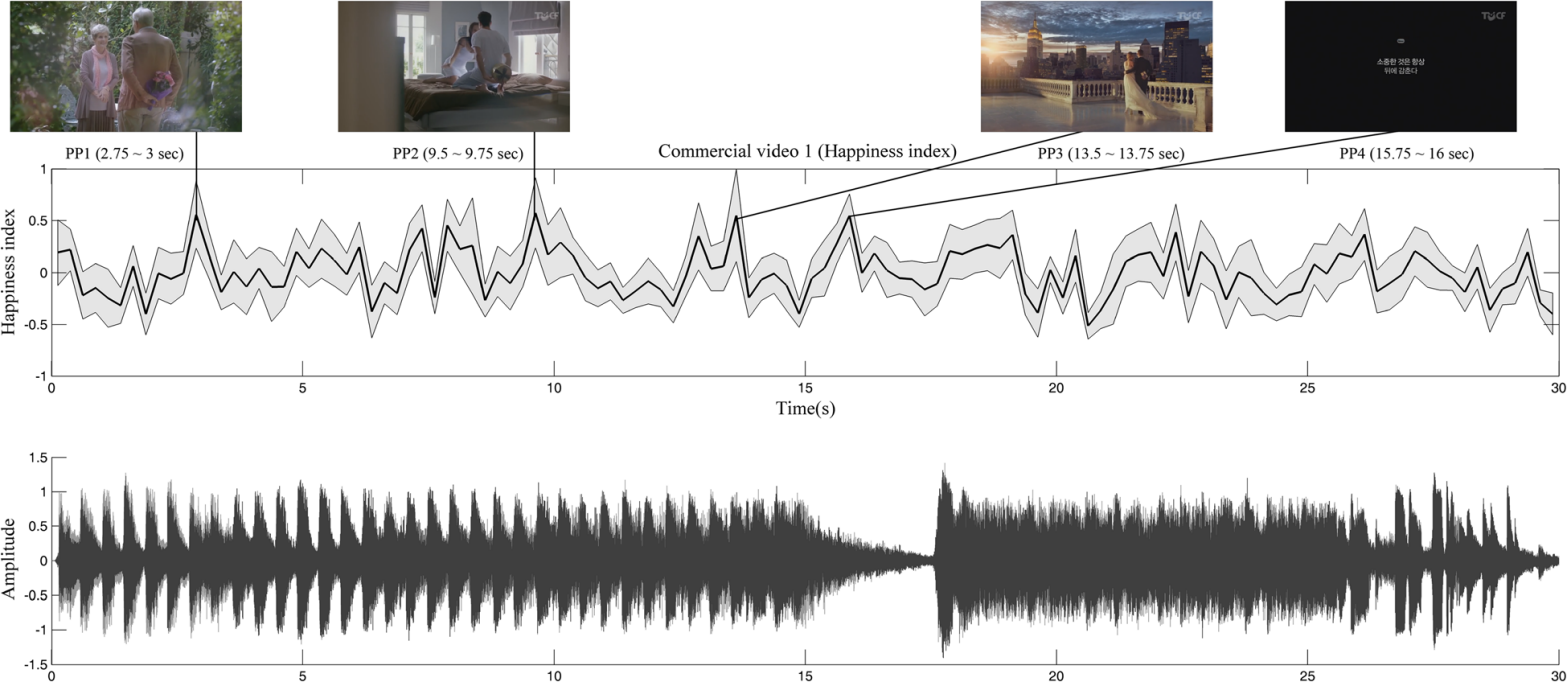
The example of the time-elapsed analysis. The time-elapsed happiness index score (mid) with scenes in four peak points (top) and audio structure (bottom).

The neurophysiological indices were analyzed as a function of elapsed time. Figure 6 shows the happiness index averaged across participants for C1 along with audio structures. From the temporal patterns of the happiness index, we chose several examples of the scenes in the TV commercial (C1), which corresponded to the average index above 0.5: 2.75 to 3 s (peak point 1 (PP1)), 9.5 to 9.75 s (peak point 2 (PP2)), 13.5 to 13.75 s (peak point 3 (PP3)), and 15.75 to 16 s (peak point 4 (PP4)). These scenes illustrated example parts of a TV commercial that mostly induced happiness emotion in participants. In particular, the fourth example showing a written message on the black background was recognized as a breaking point in terms of the audio stream where a sudden pause of music was placed, being quite different from other scenes in the commercial.

## Discussion

### EEG features for TV commercial development indices

Subject-independent features were selected using ANOVA and tenfold cross-validation. The number of feature dimensions differed by participant and index. For each participant, the regional features varied but some features were shared (Figure 2). The happiness index was related to the high-frequency band (>20 Hz) in the right fronto-central (FC5) and right temporal sites (T8). For the surprise index, the gamma band (30 to 50 Hz) in the prefrontal site (AF3) was dominant, followed by high beta and gamma bands of the left temporal site (T7), the gamma band in the occipital site (O2), and the delta band in the left frontal sites (F3 and F7). For the attention index, the high beta band of the left prefrontal site (AF3) was dominant. Previous studies [34,35] found that EEG beta activity in both temporal and parietal regions was associated with emotional valence. Other studies have also shown that temporal cortical activity was related to the distinction between positive and negative emotions [36,37]. The observed features of the present study are in line with these previous results.

### Behavioral and neurophysiological indices

Based on the behavioral data, the commercials were divided into two groups according to the ranking of behavioral indices: a ‘high-ranked’ (1 and 4) and a ‘low-ranked’ (2 and 3) commercial groups. There were no significant differences in behavioral responses within each group, but commercials differed significantly when compared between groups. As for the neurophysiological indices, the happiness index showed significant differences between commercials. C1 differed significantly from both C2 and C3 in the happiness index. C4 showed a relatively higher attention index than the commercials in the ‘low-ranked’ group. These results demonstrate that the neurophysiological indices of happiness and attention developed in this study may be used to evaluate TV commercials.

The population ratings were available in the public database we obtained our TV commercials used in the study. The population rating of each commercial (based on 5-point scale) was 3.77 for C1, 3.12 for C2, 2.91 for C3, and 3.57 for C4. Interestingly, the ranking of population ratings among these for commercials is quite consistent with the subjective ratings and neurophysiological indices in our study. This shows that the neurophysiological indices developed in a small group (18 people in our case), as validated with group behavioral responses, may be generalized to a large population [38]. It also implies that it may be possible to expect the response of population with the result from a small group of participants.

It is noteworthy that the ranking for the surprise index showed the opposite order to the other indices. Although there were no significant differences between the commercials, the surprise index order was completely reversed: C3 received the highest score while C1 received the lowest. This effect is likely due to the specific meaning of ‘surprise’ and will be covered in the last part of the ‘Discussion’ section (that is, ‘Limitations and future research’ subsection).

### Elapsed-time analysis

Each commercial can be characterized by a specific combination of scene changes, music, characters, and captions. This complicates the analysis of commercials. We therefore needed to analyze commercials as function of elapsed time. As an example for this elapsed-time analysis, we examined C1 in terms of the happiness index, that is, we analyzed the time points at which the happiness index reached the highest values during the commercial. During C1, the happiness index oscillated continuously but there were some clear peaks: PP1, PP2, PP3, and PP4. During PP1, an old couple was depicted wherein the man was hiding flowers from the woman. During PP2, a young couple was playing on the bed while another man hid in the background. During PP3, a newlywed couple and another man in hiding were presented. Considering that the other scenes have only one man or a family (father and daughter) present rather than a couple, it is plausible that happiness was induced when participants view the scenes of happy couples. The final peak point (PP4) differed slightly; the scene consisted of a black background, a smartphone, and some text. Although there were no special components in this scene, the musical stream was distinct. In the scene between 14 and 15 s - right before PP4 - there was a ‘ding’ sound (that is, similar to a bicycle horn). After this tone, sudden silence accompanied the appearance of a picture of the advertised smartphone and a message (in Korean) ‘We always hide what is precious behind us’ on a black background, after which the aforementioned sound was heard again (17 to 18 s). In the study of Gomez and Danuser [39], a relationship between musical structures and valence and arousal was discussed. Also, the relationship between auditory stimuli and emotion has been studied by others [40-42] and significant correlations have been demonstrated. These support a conjecture that the auditory structure of C1 might induce a greater happiness index.

### Applications to design advertisements

Our study proposed a neurophysiological method to evaluate TV commercials. However, it can also be used to design a TV commercial as well before its release. In particular, the design process of TV commercials may take advantage of the proposed method in the following ways: First, as our method employs simple basic stimuli irrelevant to TV commercials in the development of neurophysiological indices, one can flexibly apply this method to any commercial clips during the design stage; second, it is typically difficult to estimate the response of populations to a TV commercial in advance before its release. But the present study implies that one can use our method to evaluate a designed ad before release and predict population responses; third, the possibility of temporal analysis of video streams using our method would help designers look into individual scenes and improve their film editing, which is generally not possible with *post hoc* verbal-based methods. Hence, together with traditional survey-based methods, the proposed neurophysiological method may provide additional merits, especially for both the design and evaluation of TV commercials.

### Limitations and future research

Our study used preset video clips and a game to induce specific cognitive or emotional states. Although we used two stimuli for each emotion and selected the stimulus based on the participants’ response, the chosen stimuli might not have induced a ‘true’ emotion in the participants. Specifically, a participant might have admitted that he or she was happy even when he or she may not have actually felt as such. Furthermore, a participant may not have been in a ‘neutral’ emotional state when he or she viewed the neutral picture with nature sounds. Similarly, ‘surprise’ can have different and diverse meanings for each participant. When two people simultaneously view the same product, one of them might be surprised by the product’s functions while the other might be surprised by its shape. Therefore, stimuli for eliciting emotional and neutral states that vary less with participant characteristics are needed for future studies. Many studies have extracted neurophysiological indices for emotion from EEG data but have yielded different findings [18-22,28]. Thus, research attempting to standardize neurophysiological indices for evaluating media, including TV commercials, is necessary.

In the current study, we focused on EEG features (channels and frequency bands) but other analyses can be adopted, for example, phase synchronization or spectral coherence. A previous study [28] argued that ‘EEG and other biometric methods should not be used as a replacement of verbal-based methods, but as a supplement.’ However, it is certain that neurophysiological methods give us more information that cannot be obtained from the verbal-based methods. We presented some examples of application to design the advertisements. With the high-quality integration between different methods, neurophysiological method will show its true utility.

## Conclusions

Our study evaluated four TV commercials using neurophysiological (EEG) and conventional survey-based methods. From both measurements, two types of indices were derived: neurophysiological indices (happiness, surprise, and attention) and behavioral indices (preference rate, short-term memory rate, and recall rate). To extract the neurophysiological indices, optimal spectral features from EEG were extracted using statistical and classification models. Behavioral indices showed significant differences between commercials, especially when the commercials were divided into ‘high-ranked’ and ‘low-ranked’ groups. The corresponding neurophysiological index matched the behavioral findings. The happiness index in particular showed significant differences between ‘high-ranked’ and the ‘low-ranked’ groups. The elapsed-time analysis demonstrated that scene and auditory components affected the neurophysiological indices. Although significant differences between commercials were only found for the happiness index, the ranking of the four commercials in terms of neurophysiological indices showed a similar pattern to the behavioral ranking. The elapsed-time analysis further revealed the utility of the structural and contextual analysis method used herein. The current study demonstrates a new scheme of evaluating media content, particularly for TV commercials.
